# FgGET3, an ATPase of the GET Pathway, Is Important for the Development and Virulence of *Fusarium graminearum*

**DOI:** 10.3390/ijms252212172

**Published:** 2024-11-13

**Authors:** Caihong Liu, Lu Lei, Jing Zhu, Lirun Chen, Shijing Peng, Mi Zhang, Ziyi Zhang, Jie Tang, Qing Chen, Li Kong, Youliang Zheng, Maria Ladera-Carmona, Karl-Heinz Kogel, Yuming Wei, Pengfei Qi

**Affiliations:** 1 State Key Laboratory of Crop Gene Exploration and Utilization in Southwest China, Sichuan Agricultural University, Chengdu 611130, China; sicaulch@outlook.com; 2 Triticeae Research Institute, Sichuan Agricultural University, Chengdu 611130, China; leilu@stu.sicau.edu.cn (L.L.); zhujing@stu.sicau.edu.cn (J.Z.); lirunchen88@stu.sicau.edu.cn (L.C.); pengshijing@stu.sicau.edu.cn (S.P.); zhangmi@stu.sicau.edu.cn (M.Z.); zhangjiajia@stu.sicau.edu.cn (Z.Z.); tangjie@stu.sicau.edu.cn (J.T.); qingchen83@sicau.edu.cn (Q.C.); kongli@sicau.edu.cn (L.K.); ylzheng@sicau.edu.cn (Y.Z.); 3 Institute of Phytopathology, Research Centre for BioSystems, Land Use and Nutrition, Justus Liebig University Giessen, Heinrich-Buff-Ring 26, 35392 Giessen, Germany; maria.ladera-carmona@agrar.uni-giessen.de (M.L.-C.); karl-heinz.kogel@agrar.uni-giessen.de (K.-H.K.); 4 Institut de Biologie Moléculaire des Plantes, CNRS, Université de Strasbourg, 12 rue du Général Zimmer, 67084 Strasbourg, France

**Keywords:** *Fusarium graminearum*, Fusarium head blight, GET pathway, FgGET3, stress response, virulence

## Abstract

GET3 is an ATPase protein that plays a pivotal role in the guided entry of the tail-anchored (GET) pathway. The protein facilitates the targeting and inserting of tail-anchored (TA) proteins into the endoplasmic reticulum (ER) by interacting with a receptor protein complex on the ER. The role of GET3 in various biological processes has been established in yeast, plants, and mammals but not in filamentous fungi. *Fusarium graminearum* is the major causal agent of Fusarium head blight (FHB), posing a threat to the yield and quality of wheat. In this study, we found that FgGET3 exhibits a high degree of sequence and structural conservation with its homologs across a wide range of organisms. Ectopic expression of *FgGET3* in yeast restored the growth defects of the *Saccharomyces cerevisiae ScGET3* knock-out mutant. Furthermore, FgGET3 was found to dimerize and localize to the cytoplasm, similar to its homologs in other species. Deletion of *FgGET3* in *F. graminearum* results in decreased fungal growth, fragmented vacuoles, altered abiotic stress responses, reduced conidia production, delayed conidial germination, weakened virulence on wheat spikes and reduced DON production. Collectively, these findings underscore the critical role of *FgGET3* in regulating diverse cellular and biological functions essential for the growth and virulence of *F. graminearum*.

## 1. Introduction

Tail-anchored (TA) proteins are a distinct class of membrane-integrated proteins [1] that are involved in diverse cellular processes, including protein translocation across organelle membranes, vesicular trafficking, apoptosis, and protein quality control [2,3]. In yeast, the GET pathway is the most extensively studied pathway for targeting and inserting endoplasmic reticulum (ER)-destined TA proteins [4]. This pathway starts with the transfer of TA to a GET4/GET5/SGT2 sorting complex. GET3, an ATPase, can bind to GET4 thereby facilitating the loading of TA to GET3. Subsequently, GET3 chaperones TA proteins to the ER membrane by interacting with the ER-membrane localized GET1-GET2 receptor proteins [5,6].

GET3 is involved in multiple biological processes in different organisms, including metal homeostasis, signal transduction, and the ubiquitin-proteasome system [7]. In *Saccharomyces cerevisiae*, the knock-out (KO) of *ScGET3* leads to several seemingly unrelated phenotypes, including sensitivity to hygromycin, hydroxyurea, metal, H₂O₂, and heat stresses [5,8,9]. In *Arabidopsis thaliana*, there are three GET3 homologs; however, only AtGET3a has the conserved GET1 binding domain and is localized in the cytosol [10]. *AtGET3a* KO mutant flowers earlier than the wild-type (WT) [11]. In mammals, the deletion of *GET3* leads to embryonic lethality in mice [12], and upregulation of *GET3* has been observed in several types of human cancers [7]. In the protozoan parasite *Plasmodium falciparum*, the function of *GET3* is required in the intraerythrocytic developmental stage [13]. Homologs of GET3 are well conserved across organisms, but the functional studies of *GET3* remain poorly understood in filamentous fungi.

*Fusarium graminearum*, which is included in the top ten top 10 economically important fungal pathogens, is the predominant causal agent of Fusarium head blight (FHB) in wheat and other cereal crops [14]. The main consequence of FHB is the reduction in grained yield and quality, which affects the overall harvest and economy [15,16], and a serious threat to animal and human health by contaminating grains with mycotoxins, including the trichothecene deoxynivalenol (DON), a potent inhibitor of protein synthesis in eukaryotes [17,18]. So far, the control of FHB remains problematic. Due to the unavailability of resistant wheat cultivars, the major approach for controlling disease and limiting mycotoxin accumulation is still the use of chemical fungicides, which partly exhibit negative traits like fungicide tolerance and hazardous effects on the environment [19,20]. Therefore, there is an urgency to develop effective control strategies for FHB. Previous studies have shown that growth, development, and wheat infection of *F. graminearum* are regulated by various genes involved in different regulatory pathways [21,22,23]; however, the GET pathway associated with growth and virulence remains poorly understood. In *F. graminearum*, GET pathway genes have not been identified yet. In this study, we provide evidence that *FGSG_09891* encodes a GET3 homolog, designated as FgGET3, and we uncovered the genetic and biological functions of *FgGET3* using cellular, genetic, and biochemical approaches.

## 2. Results

### 2.1. Identification of FgGET3 in F. graminearum

**Figure 1:**
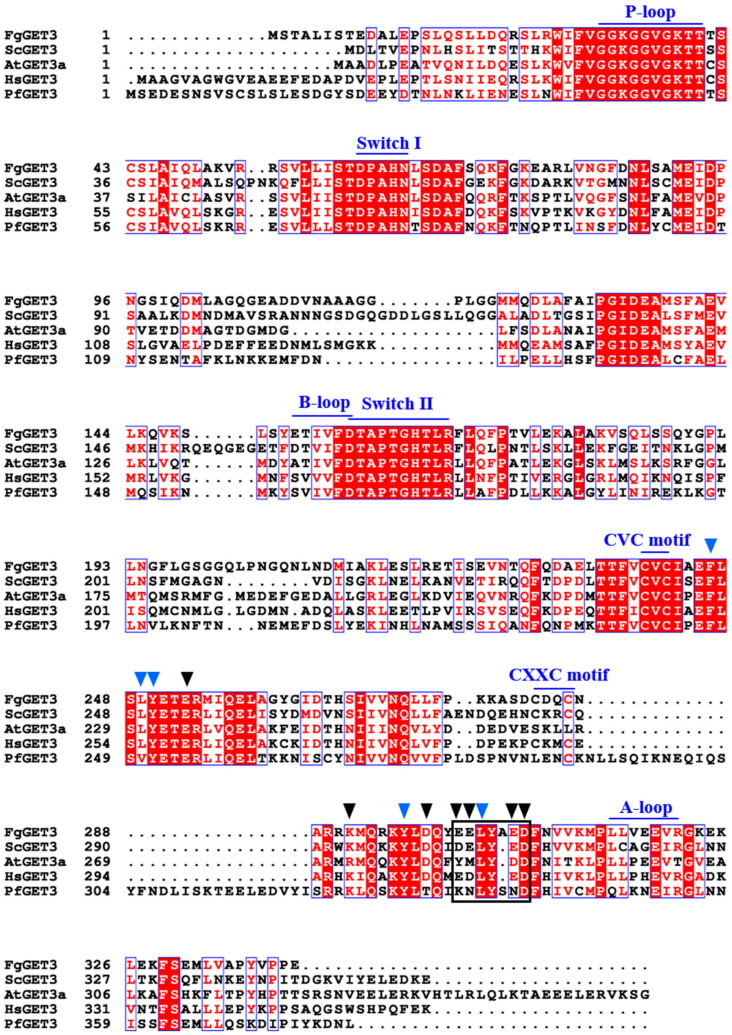
Identification of the FgGET3 protein in *Fusarium graminearum*. The aa sequence of FgGET3 (XP_011318797.1), ScGET3 (NP_010183.1) of *Saccharomyces cerevisiae*, AtGET3a (NP_563640.1) of *Arabidopsis thaliana*, HsGET3 (NP_004308.1) of *Homo sapiens* and PfGET3 (XP_001351457.1) of *Plasmodium falciparum* was aligned using Clustal W2. ESPript 3.0 was used to highlight identical (white font, highlighted in red with red), well-conserved (red font, boxed in blue) residues. The conserved motifs are labeled above with blue lines and font. The sequence involved in both the GET1-GET3 and GET2-GET3 interactions is enclosed in a black frame. The other aa involved in the GET1-GET3 interaction are indicated with blue inverted triangles above. The aa involved in the GET4-GET3 interaction are indicated with black inverted triangles.

To search for the corresponding homolog of GET3 protein in *F. graminearum*, we used the amino acid (aa) sequence of ScGET3 from *S. cerevisiae* (NP_010183.1) and queried against the *F. graminearum* database by using Basic Local Alignment Search Tool (BLAST) in NCBI database. The top hit in all queries was FGSG_09891 (XP_011318797.1), which we designated as FgGET3. FgGET3 is a 341 aa protein, sharing 47.2% identity with *S. cerevisiae* ScGET3, 50.4% identity with *A. thaliana* AtGET3a (NP_563640.1), 49.9% identity with *Homo sapiens* HsGET3 (NP_004308.1), and 42.8% identity with *P. falciparum* PfGET3 (XP_001351457.1). Domain prediction reveals that FgGET3 exhibits conserved protein motifs that are characteristic of other GET3 proteins (Figure 1), including the presence of (ⅰ) the ATPase activity-related motifs [24]; among them, the P-loop, Switch I and Switch II show conserved sequences across various species, while the B-loop and A-loop aa motif in FgGET3 is relatively conserved compared to other GET3 homologs but not entirely identical; (ⅱ) a motif that interacts with both GET1 and GET2 [25,26]; the ‘DELYED’ motif in yeast ScGET3, which can bind to ScGET1, and the RERR motif in ScGET2, which corresponds to ‘EELYAED’ in FgGET3; (ⅲ) other aa residues that facilitate interaction with GET1; specific residues in ScGET3, including F246, L249, Y250, Y298 and L305 [26], are all conserved in FgGET3; (ⅳ) the ‘CXXC’ motif, which promotes the formation of GET3 homodimers [27], corresponds to ‘CDQC’ in FgGET3; (ⅴ) aa residues involved in binding with ScGET4; in ScGET3 residues D253, K293, K297, D300, D303, E304, E307 and D308 interact with ScGET4 [28]; in FgGET3, these residues are conserved except for the substitution of Asp303 with Glu; and (ⅵ) the ‘CVC’ motif, associated with oxidative stress sensitivity [9], which is consistently conserved across different species.

### 2.2. FgGET3 Restores the Growth Defect of the Yeast ScGET3 KO Mutant

**Figure 2:**
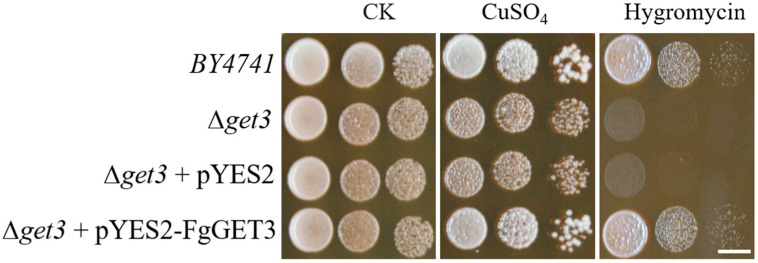
Complementation of *S. cerevisiae* strain ∆*get3* with *FgGET3*. The plasmid pYES2-FgGET3 was transformed into ∆*get3*. The cell growth (ten-fold dilutions of a starting concentration of OD_600_ = 1) without (CK) and in the presence of 3 mM CuSO_4_ at 37 °C or 200 mM hygromycin at 30 °C on YPD plates is shown. The empty pYES2 vector was transformed into ∆*get3* as a negative control. The growth of each strain was examined after 3 days of incubation. Scale bar = 5 mm.

In *S. cerevisiae*, *ScGET3* KO mutant (Δ*get3*) showed clear phenotypic changes under various stress conditions [5]. To investigate comparable function between *FgGET3* and *ScGET3*, we transformed Δ*get3* with either the empty pYES2 vector or the expression vector pYES2-FgGET3 containing the full-length cDNA of *FgGET3* and assessed their growth. As shown in Figure 2, there was no apparent difference in growth between the *S. cerevisiae* WT *BY4741* and Δ*get3* in the absence of stresses. However, when exposed to stresses such as 3 mM CuSO_4_ at 37 °C or 200 mM hygromycin at 30 °C, the Δ*get3* mutant and Δ*get3* mutant carrying the empty pYES2 vector consistently exhibited sensitive growth phenotypes. Those phenotypes were successfully rescued by the *in trans* expression of *FgGET3* to a similar level as seen in the WT *BY4741* cells. These results confirmed that *FgGET3* is a homolog of *ScGET3.*

### 2.3. FgGET3 Forms a Homodimer

**Figure 3:**
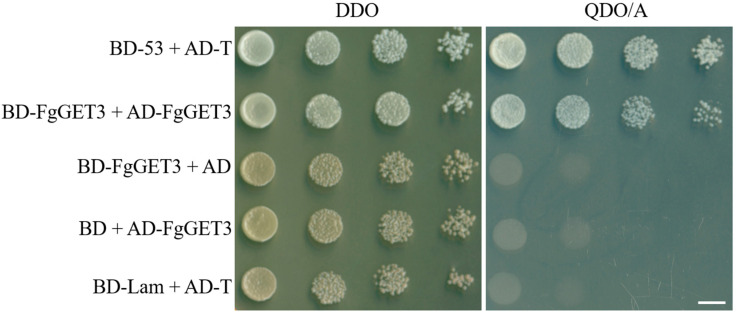
Detection of FgGET3 homodimerization using the yeast two-hybrid assay. Y2HGold strains transformed with the pair of bait and prey vectors were grown on DDO and QDO/A media (ten-fold dilutions of a starting concentration of OD_600_ = 1). The interactions of BD-53/AD-T and BD-Lam/AD-T were the positive and negative controls, respectively. AD: pGADT7; BD: pGBKT7; DDO: Double dropout medium (SD-Leu-Trp); QDO/A: Quadruple dropout medium (SD-Ade-His-Leu-Trp) supplemented with 70 ng/mL Aureobasidin A. Scale bar = 5 mm.

In *S. cerevisiae*, ScGET3 interacts with itself to form a homodimer [3,6]. To test whether a self-interaction exists in its homologs FgGET3, a yeast two-hybrid (Y2H) assay was performed. The BD-FgGET3 bait and AD-FgGET3 prey vectors were constructed and co-transformed into *Y2HGold* strains. The result showed that FgGET3 interacts with itself (Figure 3).

### 2.4. FgGET3 Localizes in the Cytoplasm

**Figure 4:**
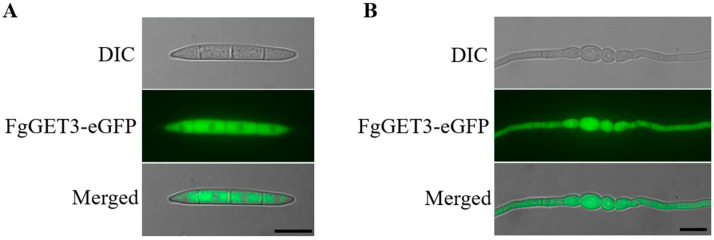
Subcellular localization of FgGET3 in (**A**) ungerminated conidia and (**B**) conidia germinating for 8 h. Scale bar = 10 µm; DIC, differential interference contrast.

Previous studies have identified that GET3 from other species localize to the cytoplasm [5,10]. Consistent with this, FgGET3 was predicted to lack a transmembrane helix (Figure S1). To verify that FgGET3 is indeed a soluble protein as predicted, we tagged the C-terminus of FgGET3 with a green fluorescent protein (eGFP) and expressed it in the WT *F. graminearum* strain. We found that FgGET3-eGFP localized to the cytoplasm in both ungerminated (Figure 4A) and germinated conidia (Figure 4B), consistent with the subcellular localization of GET3 in other species.

### 2.5. Generation of FgGET3 Mutants

To elucidate the biological function of *FgGET3* in *F. graminearum*, the KO mutant Δ*Fgget3* was created. The fragments flanking upstream and downstream of *FgGET3* were cloned into the pRF-HU2 vector, which harbored the hygromycin B phosphotransferase (*HPH*) gene. The constructed FgGET3-pRF-HU2 vector was transformed into the WT strain, and the coding sequence of *FgGET3* was replaced with the *HPH* gene through homologous recombination (Figure S2A). Transformants exhibiting resistance to hygromycin were verified via PCR analysis (Figure S2B).

The complemented strains Δ*Fgget3*-C were generated by introducing the neomycin-resistance (NEO) vector FgGET3-JM45, which encompasses a 2.1-kb region containing the gene and the promoter region, into the Δ*Fgget3* mutant. The expression of *HPH* and *NEO* genes in Δ*Fgget3*-C was detected by reverse transcription PCR (RT-PCR), demonstrating the presence of the KO and complementary vectors. Additionally, the transcript levels of *FgGET3* in the WT and Δ*Fgget3*-C strains were found to be similar, but completely absent in Δ*Fgget3* (Figure S2C). These results demonstrate successful generation of KO and complemented strains.

### 2.6. FgGET3 Is Involved in Hyphal Growth and Vacuole Fusion

**Figure 5:**
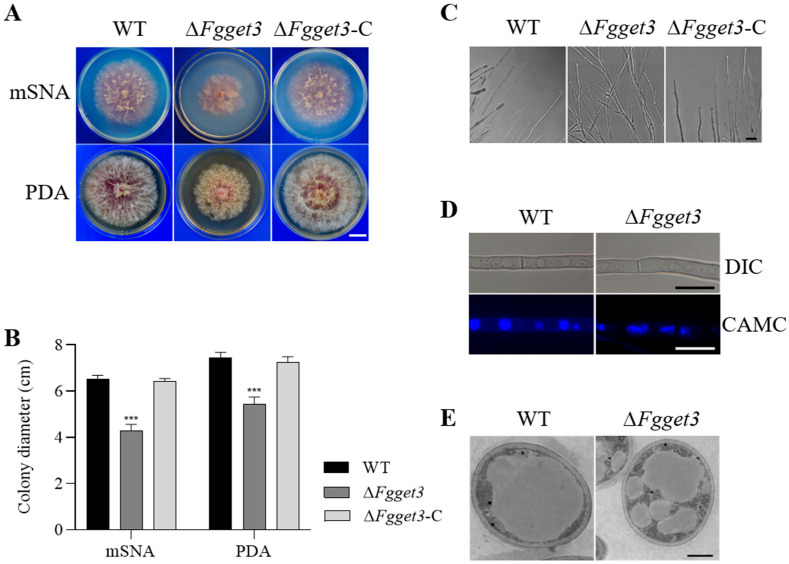
*FgGET3* in involved in vegetative growth and vacuole morphology. (**A**) Colony morphology of WT, Δ*Fgget3*, and Δ*Fgget3*-C on PDA and mSNA plates. Photos were taken after incubation at 25 °C for 4 days. Scale bar = 2 cm. (**B**) Colony diameters of indicated strains on mSNA and PDA media. Means and standard deviations were calculated from three replicates; the asterisks indicate significant differences from the WT group (Student’s *t*-test, *** *p* < 0.001). (**C**) Microscopic observation of hyphal branching patterns of WT, and Δ*Fgget3* grown on PDA medium for 2 days. Scale bar = 20 µm. (**D**) Fluorescence microscopy images of WT and Δ*Fgget3* hyphae stained with the vacuole tracker dye CMAC. DIC: differential interference contrast. Scale bar = 20 µm. (**E**) Vacuole structures in hyphae of WT and Δ*Fgget3* observed using transmission electron microscopy. Scale bar = 1 µm.

To investigate the requirement of *FgGET3* in hyphal growth, WT, Δ*Fgget3*, and Δ*Fgget3*-C strains were cultured on both nutrient-poor medium mSNA and nutrient-rich medium PDA. Compared with the WT and Δ*Fgget3*-C, the Δ*Fgget3* mutant showed a reduced vegetative growth rate on both types of media (Figure 5A,B). Further microscopic observation revealed that Δ*Fgget3* grew abnormally producing multiple hyphal branches (Figure 5C).

In addition, by staining the hyphae with the vacuole tracker 7-amino-4-chloromethylcoumarin (CAMC), we observed that Δ*Fgget3* hyphae produced fragmented small vacuoles, whereas WT cells exhibited a single large vacuole (Figure 5D). Transmission electron microscopy further confirmed the presence of fragmented vacuoles with numerous irregular spheroids in Δ*Fgget3* hyphae but not in WT (Figure 5E). These findings indicate that *FgGET3* is crucial for both hyphal growth and vacuole fusion in *F. graminearum*.

### 2.7. FgGET3 Is Involved in Fungal Responses to Various Stresses

**Figure 6:**
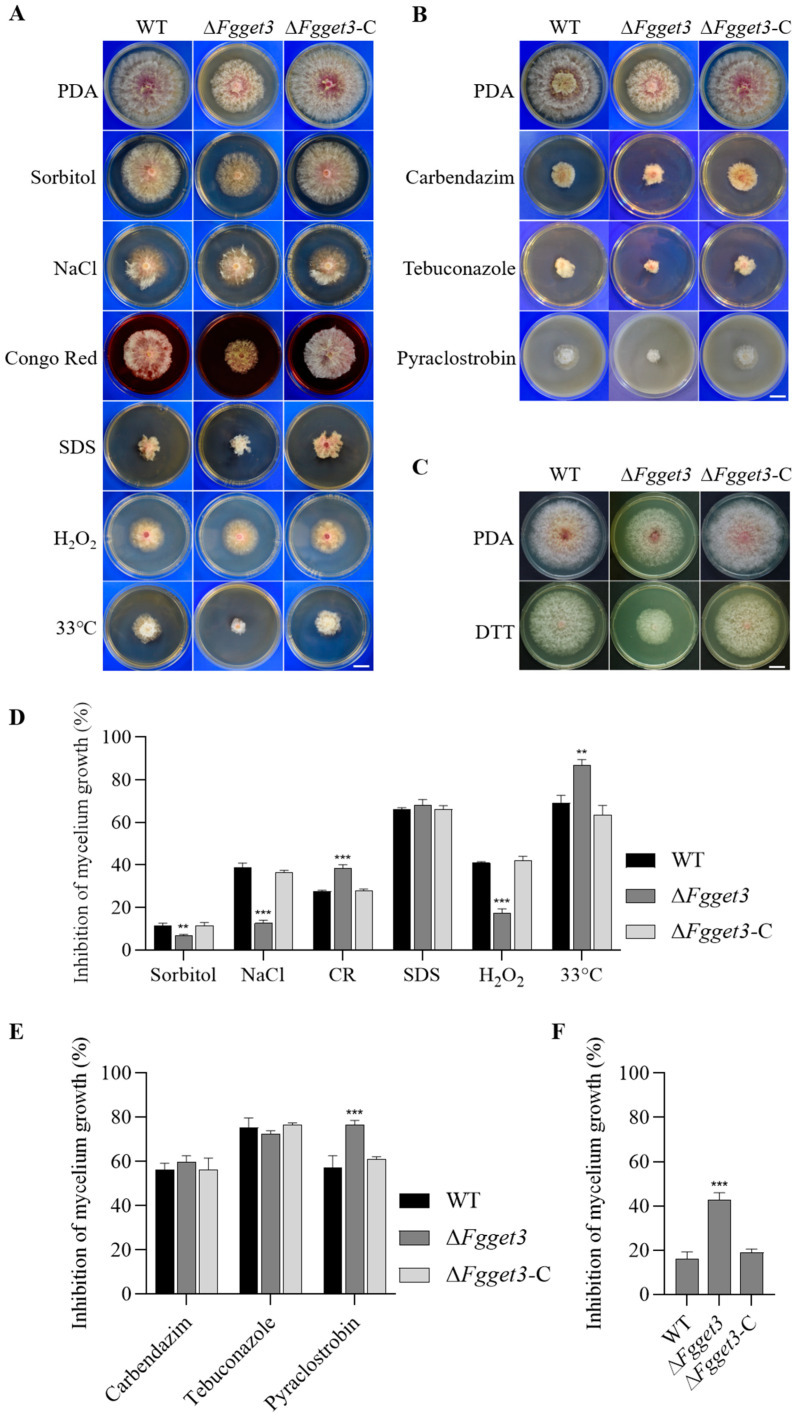
*FgGET3* impacts the responses of *F. graminearum* to various stresses. (**A**) Colony morphology of WT, Δ*Fgget3*, and Δ*Fgget3*-C in response to environmental stresses including 1M Sorbitol, 1 M NaCl, 0.5 mM Congo Red (CR), 0.025% SDS, 0.05% H_2_O_2_ and high temperature at 33 °C. Scale bar = 2 cm. (**B**) Colony morphology of indicated strains in response to fungicides including 0.4 µg/mL carbendazim, 5 µg/mL tebuconazole, and 0.6 µg/mL pyraclostrobin. Scale bar = 2 cm. (**C**) Colony morphology of WT, Δ*Fgget3*, and Δ*Fgget3*-C in response to 5 mM DTT. Scale bar = 2 cm. (**D**) Percentage of mycelium growth inhibition by environmental stresses. (**E**) Percentage of mycelium growth inhibition by fungicides. (**F**) Percentage of mycelium growth inhibition by DTT. Means and standard deviations were calculated from three replicates; the asterisks indicate significant differences from the WT group (Student’s *t*-test, ** *p* < 0.01, *** *p* < 0.001).

To investigate the function of *FgGET3* in stress responses, WT, Δ*Fgget3*, and Δ*Fgget3*-C were cultured on a PDA medium supplemented with different stress-inducing agents. Firstly, when the mycelia of strains were treated with environmental stresses, we observed that compared with WT and Δ*Fgget3*-C, Δ*Fgget3* displayed a decreased inhibition rate when treated with osmotic stress agents (Sorbitol and NaCl) and oxidative stress agents (H_2_O_2_), but was more sensitive to cell wall-perturbing agent Congo Red (CR), but not to SDS (Figure 6A,D). In addition, Δ*Fgget3* was more sensitive to high-temperature stress at 33 °C. Secondly, we determined the sensitivity of Δ*Fgget3* to metal ions, however, Δ*Fgget3* did not exhibit changed sensitivity to heavy metal ions Mg^2+^, Cu^2+^, Zn^2+^, and Fe^2+^ (Figure S3). Thirdly, in drug sensitivity assays, Δ*Fgget3* exhibited increased sensitivity to pyraclostrobin, but not to carbendazim and tebuconazole (Figure 6B,E). Fourthly, compared to that of the WT and Δ*Fgget3*-C, the Δ*Fgget3* mutant showed increased sensitivity to dithiothreitol (DTT) induced ER stress (Figure 6C,F).

### 2.8. FgGET3 Plays a Critical Role in Asexual Development

**Figure 7:**
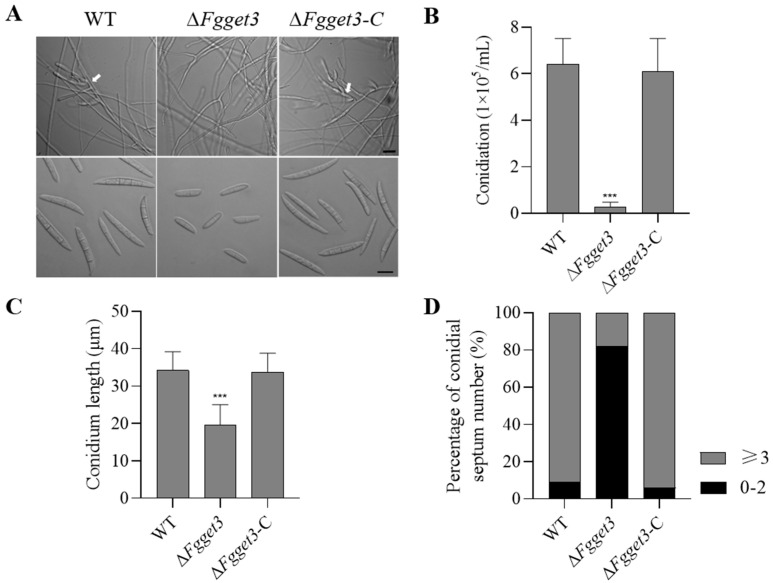

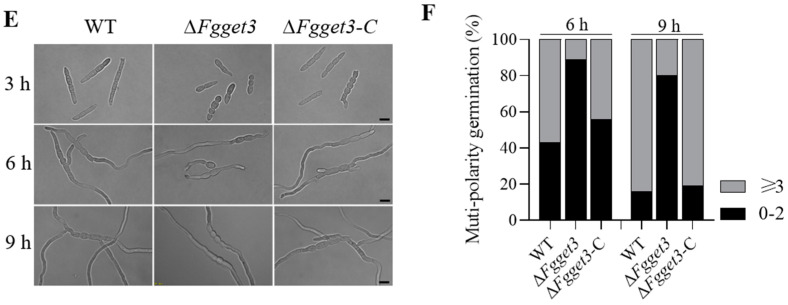
*FgGET3* is crucial for conidiation, conidial morphology, and germination of *F. graminearum*. (**A**) Morphology of phialide (white arrows) and conidia of WT, Δ*Fgget3*, and Δ*Fgget3*-C strains. Scale bar = 20 μm. (**B**) Conidiation capacity of the indicated strains in CMC liquid medium. (**C**) Conidial length of the indicated strains. Means and standard deviations were calculated from three replicates, at least 100 conidia were observed in each replicate, and the asterisks indicate significant differences from the WT group. (Student’s *t*-test, *** *p* < 0.001). (**D**) Percentages of conidia with different numbers of septa in the indicated strains. At least 300 conidia were observed from three replicates. (**E**) Morphology of germinated conidia at 3 h, 6 h, and 9 h in liquid YEPD medium. Scale bar = 20 μm. (**F**) Germination rates of the indicated strains under a microscope after 6 h and 9 h incubation in YEPD liquid medium. At least 100 conidia were randomly observed at each time point.

To investigate whether *FgGET3* is involved in conidiation, equal amounts of fresh mycelium of WT, Δ*Fgget3*, and Δ*Fgget3*-C were inoculated into a liquid CMC medium. After four days, conidia of WT and Δ*Fgget3*-C were observed forming on phialides. In contrast, these structures were rarely seen in Δ*Fgget3* (Figure 7A). Compared to those of WT and Δ*Fgget3*-C, the amount of conidia produced by Δ*Fgget3* was decreased by over 95% (Figure 7B). Microscopic examination revealed that the conidia of Δ*Fgget3* displayed morphological defects with a 40% reduction in length compared to WT and Δ*Fgget3*-C (Figure 7C). Additionally, most of the conidia of Δ*Fgget3* had two septa or fewer (82%). In contrast, conidia of WT and Δ*Fgget3*-C typically had three or more septa (91%) (Figure 7D). To explore the involvement of *FgGET3* in conidial germination, conidia were inoculated into a YEPD liquid medium. Microscopic examination revealed that, although the conidial germination of Δ*Fgget3* was not blocked completely, a significant delay was observed (Figure 7E). After 6 h, only 11% of Δ*Fgget3* conidia produced three or more germ tubes, compared to over 43% of WT and Δ*Fgget3*-C conidia. After 9 h, 20% of Δ*Fgget3* conidia produced three or more germ tubes, compared to over 81% of WT and Δ*Fgget3*-C conidia (Figure 7F). These results underscored the critical roles of *FgGET3* in conidiation, conidial morphology, and germination in *F. graminearum*.

### 2.9. FgGET3 Is Required for Virulence and DON Production

**Figure 8:**
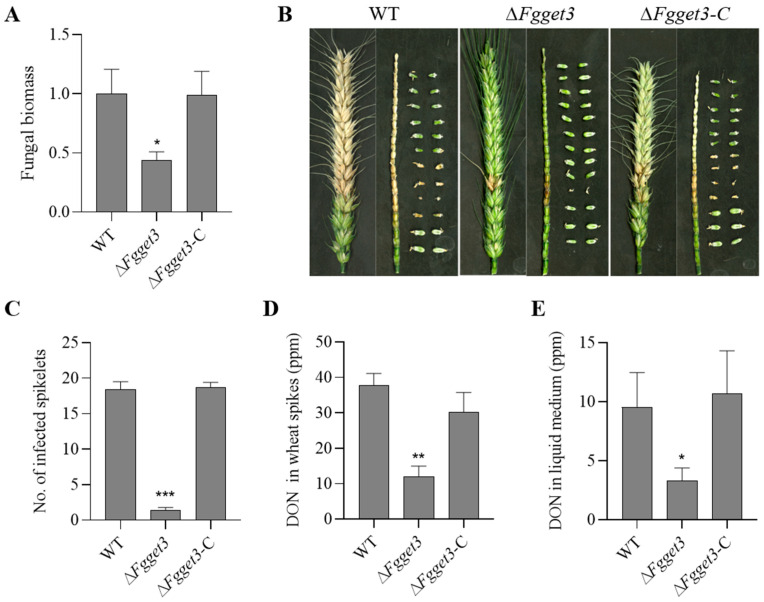
*FgGET3* contributes to the virulence and DON production of *F. graminearum*. (**A**) Fungal biomass in the inoculated spikelets was quantified using quantitative PCR (qPCR) at 2 dpi. The relative amount of fungal DNA to spikelet DNA was determined by comparing the *F. graminearum FgTUB2* gene to the wheat *TaGAPDH* gene using qPCR. The measurement was referred to as the value 1.0 obtained for the WT treatment. (**B**) The symptoms in spikelets, seeds, and rachises of infected wheat spikes infected with a conidial suspension of WT, Δ*Fgget3*, and Δ*Fgget3*-C were observed at 14 dpi. (**C**) The numbers of infected and bleached spikelets were counted at 14 dpi. (**D**) DON production in wheat spikes infected with conidia suspensions of indicated strains was quantified at 8 dpi. (**E**) The DON concentration of indicated strains was determined in a liquid medium. Means and standard deviations were calculated from three replicates; the asterisks indicate significant differences from the WT group (Student’s *t*-test, * *p* < 0.01, ** *p* < 0.005, *** *p* < 0.001).

To determine the role of *FgGET3* in the virulence of *F. graminearum*, conidia of WT, Δ*Fgget3*, and Δ*Fgget3*-C were point inoculated into flowering wheat spikes. At 2 days post-inoculation (dpi), fungal quantification in the inoculated spikes showed a 55% decrease in Δ*Fgget3* biomass compared to WT and Δ*Fgget3*-C (Figure 8A). At 14 dpi, WT and Δ*Fgget3*-C caused typical head blight symptoms in the inoculated and nearby spikelets with dark brown rachises and shriven seeds, while Δ*Fgget3* only caused limited discoloration at the inoculation site and failed to spread to adjacent rachis nodes (Figure 8B). Moreover, as DON is the most characterized virulence factor in fungal infection on wheat [29], spikelets inoculated with conidial suspensions were harvested at 8 dpi and used to assess the DON production. As the result showed (Figure 8C), compared to WT and Δ*Fgget3*-C, the DON production on infected spikes was reduced by about 68% when inoculating with Δ*Fgget3* (Figure 8D). Consistent with the wheat spikes infection results, Δ*Fgget3* also produced a much lower level (65%) of DON than WT and Δ*Fgget3*-C in a liquid medium (Figure 8E). These findings indicated that *FgGET3* contributes to the virulence and DON production of *F. graminearum.*

## 3. Discussion

Within the GET pathway, GET3 homodimeric ATPase is the central targeting factor that delivers TA proteins to the ER by interacting with the transmembrane complex [4,30]. In the present study, we identified a single GET3 homolog in *F. graminearum*, encoded by *FGSG_09891* and named FgGET3. FgGET3 exhibits high aa sequence identity with GET3 homologs from other eukaryotes and possesses a conserved protein sequence, including ATPase activity-related motifs, interaction sites with GET1, GET2, GET4, and conserved aa sites for dimerization (Figure 1). Functional complementation in *S. cerevisiae* demonstrated that *FgGET3* can restore the function of *ScGET3* in yeast (Figure 2), indicating its conserved gene function. Yeast two-hybrid assays confirmed that FgGET3 forms homodimers (Figure 3). Moreover, FgGET3 is predicted to lack transmembrane domains (Figure S1) and further confirmed to localize to the cytoplasm in conidia and hyphae as shown by eGFP fusion expression analysis (Figure 4), consistent with GET3 homologs in other species. Our results confirmed that FgGET3 is indeed a GET3 functional homolog in *F. graminearum*.

In *S. cerevisiae*, the deletion of *ScGET3* had no significant impact on vegetative growth [5]. In contrast, the Δ*Fgget3* mutant exhibited impaired vegetative growth on both nutrient-poor and nutrient-rich media, accompanied by increased hyphal branching (Figure 5A). Additionally, Δ*Fgget3* exhibited defects in conidial reproduction, morphology, and germination (Figure 7). *ScGET3* plays a role in mediating the responses of *S. cerevisiae* to different environmental stress conditions; its KO mutant Δ*get3* showed increased sensitivity to cell-wall stress [31], oxidative stress [9], elevated temperature stress, and metal stress [5]. Similarly, Δ*Fgget3* was sensitive to cell wall stress and elevated temperature (Figure 6A). However, Δ*Fgget3* showed enhanced tolerance to osmotic stress (Figure 6A) and no impact on metal stress (Figure S2). Furthermore, Δ*Fgget3* showed increased tolerance to osmotic stress (Figure 6A). Given the observed defect in vacuole morphology (Figure 5D,E), it is reasonable to speculate that Δ*Fgget3* exhibited an altered response to various environmental stresses. These discrepancies suggest that GET3 proteins may have partly distinct roles among different fungi.

In *F. graminearum*, virulence is a complex mechanism regulated by multiple factors [21,22]. On flowering wheat spikes, Δ*Fgget3* produced less fungal biomass in the early stages of infection and failed to spread through the rachis (Figure 8), possibly due to defects in both mycelial and conidial development stages that could potentially influence virulence. In addition, DON is considered another key pathogenicity factor in *F. graminearum* [32,33]. The total amount of DON in Δ*Fgget3* was reduced both on infected spikes and in liquid medium (Figure 8), which could be an additional factor contributing to the impaired infection capability of *F. graminearum*. Currently, the application of chemical fungicides is still one main approach for controlling FHB. Interestingly, Δ*Fgget3* showed higher sensitivity to pyraclostrobin (Figure 6), suggesting that *FgGET3* may be a potential target for this fungicide.

Deletion of *ScGET3* in yeast leads to various growth defects, possibly due to the mislocalization or aggregation of TA proteins near the ER [5]. In *A. thaliana*, the KO of *AtGET3* resulted in increased sensitivity of seedlings to the ER stressor DTT [11]. Similarly, the KO of *FgGET3* in *F. graminearum* also exhibited increased sensitivity to DTT (Figure 6), which may indicate aggregation of TA proteins in the cytoplasm upon gene deletion. Hence, the multifaceted defects caused by the loss of *FgGET3* function in *F. graminearum* may also be attributed to the impaired functionality of TA proteins and thus require further validation.

## 4. Materials and Methods

### 4.1. Fungal Strains and Growth Conditions

*F. graminearum* strain DAOM180378 (Canadian Fungal Culture Collection, AAFC, 270 Ottawa, ON, Canada) was used as the parental WT. The colony characteristics and growth rate of both the WT and transformants were examined on Potato Dextrose Agar (PDA) plates (Aobox, Beijing, China) and modified Synthetischer Nährstoffarmer Agar (mSNA) medium, and further cultured at 25 °C for 4 days under dark conditions. The radial growth of mycelium was measured as previously described [34]. To assess the sensitivity of mycelium to stresses, 5 mm mycelial plugs of each strain were inoculated onto PDA plates containing various stressors at concentrations indicated in the figure legends. The percentage of inhibition of mycelial radial growth was calculated as described by [35]. Each treatment was carried out in at least five Petri dishes and the experiments were repeated three times.

For the evaluation of conidiation, 20 mg of fresh mycelium taken from the periphery of a 3-day-old colony was cultured in a 150 mL flask containing 50 mL of carboxymethylcellulose (CMC) medium [36]. The cultures were shaken at 180 rpm at 25 °C for 4 days and the number of conidia in each flask was counted using a hemocytometer (Sigma-Aldric, St. Louis, MO, USA). The conidial morphology and the number of septa were observed using an Olympus-BX63 fluorescence microscope (DP80; Olympus, Tokyo, Japan), and the colonial length and width were measured using the Olympus CellSens Dimension software ver.1.18 (Olympus, Tokyo, Japan). Germination of conidia was determined by culturing 1 × 10^5^ conidia of each strain in 5 mL yeast extract peptone dextrose (YEPD) liquid medium [37] and incubating at 25 °C on a rotary shaker. The germ tubes per 100 total conidia were counted at 3 h, 6 h, and 9 h after inoculation.

### 4.2. Sequence Analysis

All the sequence data used in this study can be found in the National Center for Biotechnology Information (NCBI, https://www.ncbi.nlm.nih.gov/) (accessed on 20 August 2024). The detailed accession numbers for FgGET3 and its homologs from various species used in this study are as follows: *F. graminearum* (XP_011318797.1), *S. cerevisiae* ScGET3 (NP_010183.1), *A. thaliana* AtGET3a (NP_563640.1), *H. sapiens* HsGET3 (NP_004308.1), and *P. falciparum* PfGET3 (XP_001351457.1). Clustal W2 was used to generate amino acid sequence comparisons.

### 4.3. Fungal Transformation

The KO mutant of FgGET3 was generated using a homologous recombination strategy to replace *FgGET3* with a hygromycin B phosphotransferase gene (*HPH*) gene. For this purpose, a gene replacement vector was first constructed. 748 bp upstream (UP) and 550 bp downstream (Down) flanking sequences of *FgGET3* were amplified by PCR using the genomic DNA of WT, and the resulting amplicons were fused to the pRF-HU2 vector [38] with ClonExpress II One Step Cloning Kit (Vazyme, Nanjing, China). The constructed FgGET3-pRF-HU2 vector was transformed into WT by the *Agrobacterium tumefaciens*-mediated transformation method [39]. Putative KO transformants were selected with 100 μg/mL hygromycin, and confirmed by PCR assays. To complement the Δ*Fgget3* mutation, a PCR product, including the native promoter and open reading fragment of the gene, was cloned into the vector JM45 that contains a neomycin resistance gene. The constructed vector was transformed into the protoplast of the KO mutant by the polyethylene glycol (PEG)-mediated fungal transformation method [40]. Putative complementary transformants were selected by 100 μg/mL neomycin and further identified by PCR and RT-PCR assay. For generating the FgGET3-eGFP fusion construct, the coding sequence of *FgGET3* was amplified and inserted into the vector pRFHUE-eGFP [41], and the resulting construct pRFHUE-eGFP-FgGET3 was transformed into the WT strain by *A. tumefaciens*-mediated transformation. FgGET3-GFP transformants were selected with hygromycin and identified by PCR. All primers used are listed in Table S1.

### 4.4. Virulence and DON Production Assays

The wheat (*Triticum aestivum*) cultivar SM482, which is highly susceptible to *F. graminearum*, was used for the virulence tests. The plants were grown as previously described [36]. Conidia of WT, Δ*Fgget3*, and Δ*Fgget3*-C were collected from a CMC medium and adjusted to a concentration of 1 × 10^5^ conidia in 1 mL of sterile water. The floral cavity of the outer florets of the 5th spikelet from the bottom of the wheat spike was inoculated with 10 μL of conidial suspension at each point. After inoculation, the spikes were covered with a plastic bag for 48 h to maintain high humidity. The infected florets from five spikes were harvested at 48 h post-inoculation to estimate the relative fungal biomass within the plants. Following the removal of the plastic bags, the wheat spikelets were cultivated for an additional 12 days to evaluate typical FHB symptoms. The experiments were repeated three times with each repetition including at least ten replicates for each fungal strain.

To measure DON production in wheat spikes, conidial suspensions of WT, Δ*Fgget3*, and Δ*Fgget3*-C strains were injected into each floret of the whole spikes. There were at least five spikes for each strain. The infected spikes were collected at 8 dpi and ground to a fine powder in liquid nitrogen. To test the DON production in the liquid medium, a two-stage protocol was employed as previously described [32]. The amount of DON was quantified by using a competitive ELISA-based DON detection kit (Mlbio, Shanghai, China) and a Multiskan Spectrum instrument (Thermo Fisher Scientific, Waltham, MA, USA).

### 4.5. Nucleic Acid Extraction and PCR Assay

The genomic DNA from mycelium and plants was extracted using the DNA extraction kit (Biofit, Chengdu, China). Total RNA was extracted with Trizol Reagent (Thermo Fisher Scientific), and further used to perform reverse transcription to generate First-strand cDNA using HiScript III 1st Strand cDNA Synthesis Kit (Vazyme, Nanjing, China).

To measure the relative amount of fungal biomass in infected wheat spikelets, total DNA was extracted, and the β-tubulin gene *FgTUB2* (*FGSG_09530*) of *F. graminearum* was quantified relative to the *TaGAPDH* gene of *Triticum aestivum* using quantitative PCR (qPCR) [42]. The qPCR was run on the MyiQ Real-Time PCR Detection System (Bio-Rad, Hercules, CA, USA) with the Taq Pro Universal SYBR qPCR Master Mix (Vazyme, Nanjing, China). The relative quantification was calculated by the 2^−ΔΔCT^ method [43] with three biological replicates for each treatment. The primers used throughout the paper are listed in Table S1.

### 4.6. Staining and Microscopic Examination

For observing the hyphae morphology, fresh mycelia of WT and Δ*Fgget3* taken from 2-day-old colonies on PDA plates were fixed with 2.5% (*v*/*v*) glutaraldehyde. Subsequently, the mycelia were dehydrated in a graded series of ethanol and embedded in Epon812 (Sigma-Aldric, St. Louis, MO, USA). After cutting the ultrathin section with an ultramicrotome EM UC7 (Leica, Weztlar, Germany), specimens were stained with uranyl acetate and lead citrate, and then the hyphal morphology was observed with an HT7700 120 KV transmission electron microscopy (Hitachi, Tokyo, Japan). For vacuole morphology examination, hyphae of WT and Δ*Fgget3* were stained with 7-amino-4-chloromethylcoumarin (CAMC) [44]. To observe the expression of *FgGET3*, conidia of the *FgGET3-eGFP* strain were cultured in YEPD medium for 8 h, and the GFP fluorescence was observed using an Olympus-BX63 fluorescence microscope (DP80; Olympus, Tokyo, Japan) with the GFP filter set (excitation 488 nm, emission 520 nm).

### 4.7. Yeast Complementation Assay

*S. cerevisiae* strains, WT strain *BY4741*, and ∆*get3* mutant (YDL100C) were obtained from HORIZON (Dublin, Ireland). The full length of *FgGET3* was amplified from the cDNA of WT and subsequently cloned into the pYES2 vector (Thermo Fisher Scientific) to construct the pYES2-FgGET3 vector. The pYES2-*FgGET3* vector was transformed into ∆*get3* using the lithium acetate method according to the manufacturer’s instructions (Thermo Fisher Scientific). At the same time, the empty pYES2 vector was transformed into the *BY4741* and ∆*get3* strains as controls. The uracil-deficient selective medium was used to select the transformants (Clontech, Mountain View, CA, USA). Transformants exhibiting uracil prototrophy were then diluted in distilled water, and aliquots of 10 μL were plated in 10-fold serial dilutions on YPD medium (10 g yeast extract, 20 g peptone, 20 g glucose, 10 g agar per liter) supplemented with chemicals including hydroxyurea, hygromycin and CuSO_4_ at concentrations as indicated in figure legends.

### 4.8. Yeast Two-Hybrid Assay

To verify whether FgGET3 forms a homodimer, the cDNA of the *FgGET3* gene was inserted into both the prey vector pGADT7 (AD) and bait vector pGBKT7 (BD) (Clontech, Mountain View, CA, USA). The pair of AD-FgGET3 and BD-FgGET3 vectors were co-transformed into *S. cerevisiae* strain *Y2HGold* according to the manufacturer’s instructions (Clontech, Mountain View, CA, USA). The growth of the transformants was determined on both DDO (Double dropout medium: SD-Leu/Trp) and QDO/X/A (Quadruple dropout medium: SD/-Ade/-His/-Leu/-Trp supplemented with Aureobasidin A) medium for 3 days. The AD-T/BD-53 pair and AD-T/BD-Lam pair were used as the positive and negative control, respectively.

### 4.9. Statistical Analyses

All data analysis was performed using GraphPad 8.0 and graphs are shown as means ± standard deviation (SD). The significant differences between experimental and control groups were determined using the two-tailed Student’s *t*-test, and all experiments in this study were performed with at least three replicated measurements.

## 5. Conclusions

Our results indicate that *FgGET3* plays a pleiotropic role in the *F. graminearum* disease cycle, involving mycelium growth, stress response, asexual development, and plant infection. Understanding the biological roles of *FgGET3* in impacting the plant disease cycle provides fundamental new knowledge for the development of novel methods of disease intervention methods. Further insight into the role of *FgGET3* in TA protein targeting and inserting could help elucidate its cellular function and how it regulates.

## Data Availability

The data that support the findings of this study are available from the corresponding author upon reasonable request.

## Supplementary Materials

The following supporting information can be downloaded at https://www.mdpi.com/article/10.3390/ijms252212172/s1.

## References

[1] Kutay U., Hartmann E., Rapoport T.A. (1993). A class of membrane proteins with a C-terminal anchor. Trends Cell Biol..

[2] Hegde R.S., Keenan R.J. (2011). Tail-anchored membrane protein insertion into the endoplasmic reticulum. Nat. Rev. Mol. Cell Biol..

[3] Chartron J.W., Clemons W.M., Suloway C.J.M. (2012). The complex process of GETting tail-anchored membrane proteins to the ER. Curr. Opin. Struct. Biol..

[4] Mateja A., Keenan R.J. (2018). A structural perspective on tail-anchored protein biogenesis by the GET pathway. Curr. Opin. Struct. Biol..

[5] Schuldiner M., Metz J., Schmid V., Denic V., Rakwalska M., Schmitt H.D., Schwappach B., Weissman J.S. (2008). The GET complex mediates insertion of tail-anchored proteins into the ER membrane. Cell.

[6] Wang F., Whynot A., Tung M., Denic V. (2011). The mechanism of tail-anchored protein insertion into the ER membrane. Mol. Cell..

[7] Simpson P.J., Schwappach B., Dohlman H.G., Isaacson R.L. (2010). Structures of get3, get4, and get5 provide new models for TA membrane protein targeting. Structure.

[8] Shen J., Hsu C.M., Kang B.K., Rosen B.P., Bhattacharjee H. (2003). The *Saccharomyces cerevisiae Arr4p* is involved in metal and heat tolerance. Biometals.

[9] Voth W., Schick M., Gates S., Li S., Vilardi F., Gostimskaya I., Southworth D.R., Schwappach B., Jakob U. (2014). The protein targeting factor Get3 functions as ATP-independent chaperone under oxidative stress conditions. Mol. Cell.

[10] Xing S., Mehlhorn D.G., Wallmeroth N., Asseck L.Y., Kar R., Voss A., Denninger P., Schmidt V.A.F., Schwarzländer M., Stierhof Y.D. (2017). Loss of GET pathway orthologs in *Arabidopsis thaliana* causes root hair growth defects and affects SNARE abundance. Proc. Natl. Acad. Sci. USA.

[11] Srivastava R., Zalisko B.E., Keenan R.J., Howell S.H. (2017). The GET system inserts the tail-anchored protein, SYP72, into endoplasmic reticulum membranes. Plant Physiol..

[12] Mukhopadhyay R., Ho Y.S., Swiatek P.J., Rosen B.P., Bhattacharjee H. (2006). Targeted disruption of the mouse *Asna1* gene results in embryonic lethality. Febs Letters.

[13] Kumar T., Maitra S., Rahman A., Bhattacharjee S. (2021). A conserved guided entry of tail-anchored pathway is involved in the trafficking of a subset of membrane proteins in *Plasmodium falciparum*. PLoS Pathog..

[14] Dean R., Van Kan J.A.L., Pretorius Z.A., Hammond-Kosack K.E., Di Pietro A., Spanu P.D., Rudd J.J., Dickman M., Kahmann R., Ellis J. (2012). The top 10 fungal pathogens in molecular plant pathology. Mol. Plant Pathol..

[15] Figueroa M., Hammond-Kosack K.E., Solomon P.S. (2018). A review of wheat diseases-a field perspective. Mol. Plant Pathol..

[16] Powell A.J., Vujanovic V. (2021). Evolution of Fusarium head blight management in wheat: Scientific perspectives on biological control agents and crop genotypes protocooperation. Appl. Sci..

[17] Van Egmond H.P., Schothorst R.C., Jonker M.A. (2007). Regulations relating to mycotoxins in food: Perspectives in a global and European context. Anal. Bioanal. Chem..

[18] Trail F. (2009). For Blighted Waves of Grain: *Fusarium graminearum* in the postgenomics era. Plant Physiol..

[19] Kazan K., Gardiner D.M., Manners J.M. (2012). On the trail of a cereal killer: Recent advances in *Fusarium graminearum* pathogenomics and host resistance. Mol. Plant Pathol..

[20] Chen A.H., Islam T., Ma Z.H. (2022). An integrated pest management program for managing Fusarium head blight disease in cereals. J. Integr. Agric..

[21] Niu G., Yang Q., Liao Y., Sun D., Tang Z., Wang G., Xu M., Wang C.W., Kang J.G. (2024). Advances in understanding *Fusarium graminearum*: Genes involved in the regulation of sexual development, pathogenesis, and deoxynivalenol biosynthesis. Genes.

[22] Xu M., Wang Q., Wang G., Zhang X., Liu H., Jiang C. (2022). Combatting Fusarium head blight: Advances in molecular interactions between *Fusarium graminearum* and wheat. Phytopathol. Res..

[23] Rampersad S.N. (2020). Pathogenomics and management of Fusarium diseases in plants. Pathogens.

[24] Leipe D.D., Wolf Y.I., Koonin E.V., Aravind L. (2002). Classification and evolution of P-loop GTPases and related ATPases. J. Mol. Biol..

[25] Mariappan M., Mateja A., Dobosz M., Bove E., Hegde R.S., Keenan R.J. (2011). The mechanism of membrane-associated steps in tail-anchored protein insertion. Nature.

[26] Stefer S., Reitz S., Wang F., Wild K., Pang Y.Y., Schwarz D., Bomke J., Hein C., Löhr F., Bernhard F. (2011). Structural basis for tail-anchored membrane protein biogenesis by the Get3-receptor complex. Science.

[27] Mateja A., Szlachcic A., Downing M.E., Dobosz M., Mariappan M., Hegde R.S., Keenan R.J. (2009). The structural basis of tail-anchored membrane protein recognition by Get3. Nature.

[28] Gristick H.B., Rao M., Chartron J.W., Rome M.E., Shan S.O., Clemons W.M. (2014). Crystal structure of ATP-bound Get3-Get4-Get5 complex reveals regulation of Get3 by Get4. Nat. Struct. Mol. Biol..

[29] Boenisch S., Boenisch M.J., Schäfer W. (2011). *Fusarium graminearum* forms mycotoxin producing infection structures on wheat. BMC Plant Biol..

[30] Mateja A., Paduch M., Chang H.Y., Szydlowska A., Kossiakoff A.A., Hegde R.S., Keenan R.J. (2015). Structure of the Get3 targeting factor in complex with its membrane protein cargo. Science.

[31] Brown J.A., Sherlock G., Myers C.L., Burrows N.M., Deng C., Wu H.I., McCann K.E., Troyanskaya O.G., Brown J.M. (2006). Global analysis of gene function in yeast by quantitative phenotypic profiling. Mol. Syst. Biol..

[32] Jiang C., Zhang C., Wu C., Sun P., Hou R., Liu H., Wang C., Xu J.R. (2016). *TRI6* and *TRI10* play different roles in the regulation of deoxynivalenol (DON) production by CAMP signaling in *Fusarium graminearum*. Environ. Microbiol..

[33] Chen Y., Kistler H.C., Ma Z. (2019). Annual review of phytopathology *Fusarium graminearum* trichothecene mycotoxins: Biosynthesis, regulation, and management. Annu. Rev. Phytopathol..

[34] Qi P.F., Zhang Y.Z., Liu C.H., Zhu J., Chen Q., Guo Z.R., Wang Y., Xu B.J., Zheng T., Jiang Y.F. (2018). *Fusarium graminearum* ATP-binding cassette transporter gene *FgABCC9* is required for its transportation of salicylic acid, fungicide resistance, mycelial growth and pathogenicity towards wheat. Int. J. Mol. Sci..

[35] Qi P.F., Johnston A., Balcerzak M., Rocheleau H., Harris L.J., Long X.Y., Wei Y.M., Zheng Y.L., Ouellet T. (2012). Effect of salicylic acid on *Fusarium graminearum*, the major causal agent of fusarium head blight in wheat. Fungal Biol..

[36] Yun Y., Liu Z., Yin Y., Jiang J., Chen Y., Xu J.R., Ma Z. (2015). Functional analysis of the *Fusarium graminearum* phosphatome. New Phytol..

[37] Hou Z., Xue C., Peng Y., Katan T., Corby Kistler H., Xu J.R. (2002). A mitogen-activated protein kinase gene (*MGV1*) in *Fusarium graminearum* is required for female fertility, heterokaryon formation, and plant infection. Mol. Plant Microbe Interact..

[38] Frandsen R.J.N., Andersson J.A., Kristensen M.B., Giese H. (2008). Efficient four fragment cloning for the construction of vectors for targeted gene replacement in filamentous fungi. BMC Mol. Biol..

[39] Michielse C.B., Hooykaas P.J.J., van den Hondel C.A.M.J.J., Ram A.F.J. (2005). *Agrobacterium*-mediated transformation as a tool for functional genomics in fungi. Curr. Genet..

[40] Catlett N.L., Lee B.N., Yoder O.C., Turgeon B.G. (2003). Split-marker recombination for efficient targeted deletion of fungal genes. Fungal Genet. Rep..

[41] Crespo-Sempere A., López-Pérez M., Martínez-Culebras P.V., González-Candelas L. (2011). Development of a green fluorescent tagged strain of *Aspergillus Carbonarius* to monitor fungal colonization in grapes. Int. J. Food Microbiol..

[42] Chen Q., Lei L., Liu C.H., Zhang Y.Z., Xu Q., Zhu J., Guo Z., Wang Y., Li Q.C., Li Y. (2021). Major facilitator superfamily transporter gene *FgMFS1* is essential for *Fusarium graminearum* to deal with salicylic acid stress and for its pathogenicity towards wheat. Int. J. Mol. Sci..

[43] Livak K.J., Schmittgen T.D. (2001). Analysis of relative gene expression data using real-time quantitative PCR and the 2(-Delta Delta C(T)) Method. Methods.

[44] Shoji J.Y., Arioka M., Kitamoto K. (2006). Vacuolar membrane dynamics in the filamentous fungus *Aspergillus oryzae*. Eukaryot. Cell.

