# Supplementary material for: FgGET3, an ATPase of the GET Pathway, Is Important for the Development and Virulence of Fusarium graminearum
Source: Int J Mol Sci. 2024 Nov 13;25(22):12172. doi: 10.3390/ijms252212172 (PMC11594295; doi:10.3390/ijms252212172)
Supplement: Supplementary file 1 [file ijms-25-12172-s001.zip › ijms-3297581-supplementary.pdf]

Supplementary Materials

XP_011318797.1

```
1   MSTALISTED ALEPSLQSLL DQRSLRWIFV GGKGGVGKTT TSCSLAIQLA KVRRSVLLIS
61  TDPAHNLSDA FSQKFGKEAR LVNGFDNLSA MEIDPNGSIQ DMLAGQGEAD DVNAAAGGPL
121 GGMMQDLAFA IPGIDEAMSF AEVLKQVKSL SYETIVFDTA PTGHTLRFLQ FPTVLEKALA
181 KVSQLSSQYG PLLNGFLGSG GQLPNGQNLN DMIAKLESLR ETISEVNTQF QDAELTTFVC
241 VCIAEFLSLY ETERMIQELA GYGIDTHSIV VNQLLFPKKA SDCDQCNARR KMQRKYLDQY
301 EELYAEDFNV VKMPLLVEEV RGKEKLEKFS EMLVAPYVPP E
```

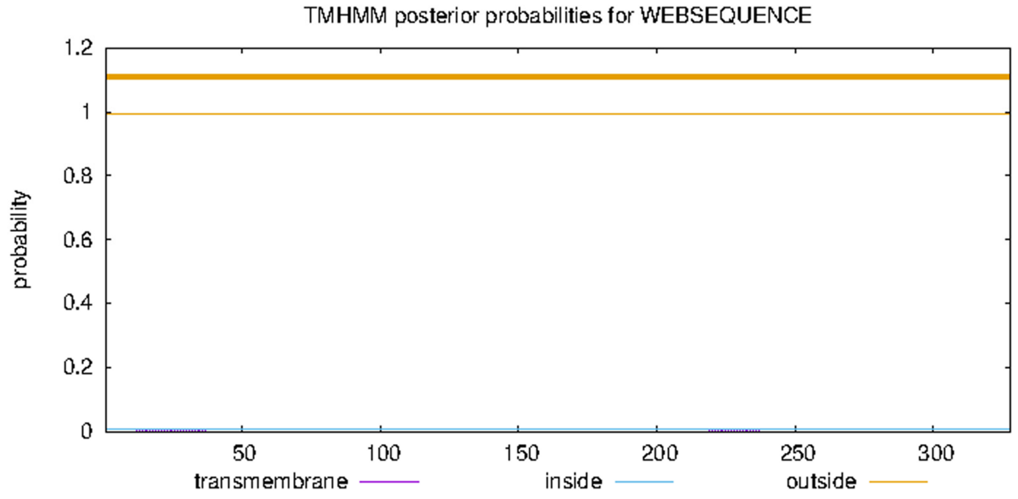

Figure S1. Analysis of FgGET3 (XP_011318797.1) protein sequence. Plot below shows the posterior probabilities for transmembrane helices according to the TMHMM Server v.2.0 (http://www.cbs.dtu.dk/services/TMHMM/).

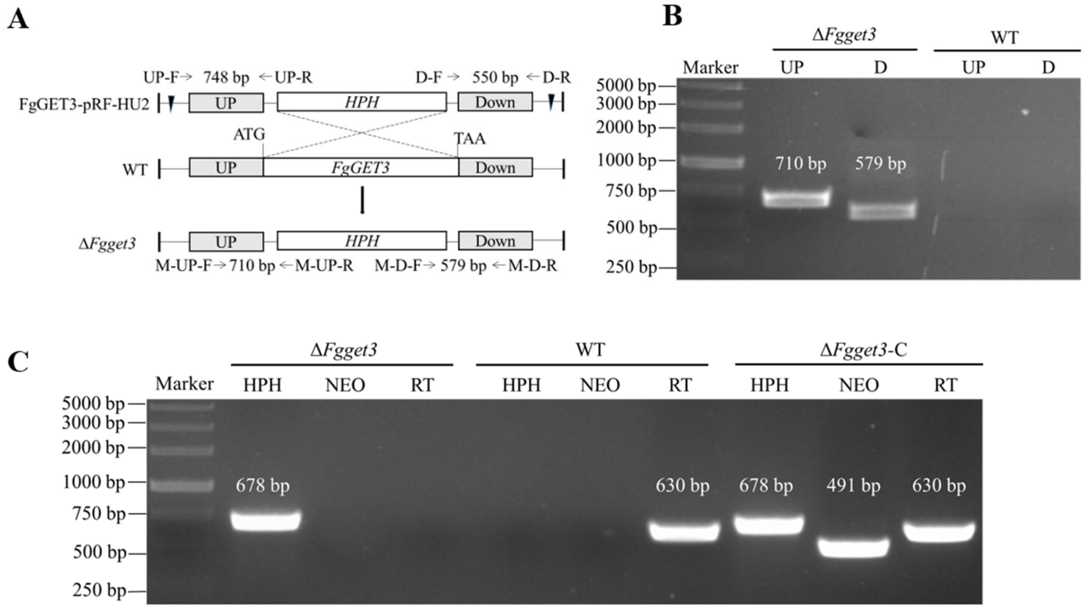

Figure S2. Construction of Δ*Fgget3* and Δ*Fgget3*-C mutants. (**A**) Scheme of constructs and gene structures for the disruption of *FgGET3*. The 748 bp upstream (UP) and 550 bp downstream (Down) flanking sequences from the DNA of the wild-type (WT) strain were amplified using primer pairs UP-F/R and D-F/R, respectively, then they were cloned into the empty pRF-HU2 vector to construct the knock-out vector FgGET3-pRF-HU2. Δ*Fgget3* mutants were generated by replacing the *FgGET3* gene in WT with the *Hygromycin phosphotransferase* (*HPH*) gene. (**B**) PCR verification of Δ*Fgget3* with primer pairs M-UP-F/R and M-D-F/R. (**C**) Reverse transcription PCR was performed to verify the expression of hygromycin-resistance, neomycin-resistance genes and *FgGET3* in Δ*Fgget3*, WT and Δ*Fgget3*-C using primers HPH-F/R(HPH), NEO-F/R (NEO) and RT-FgGET3-F/R (RT), respectively.

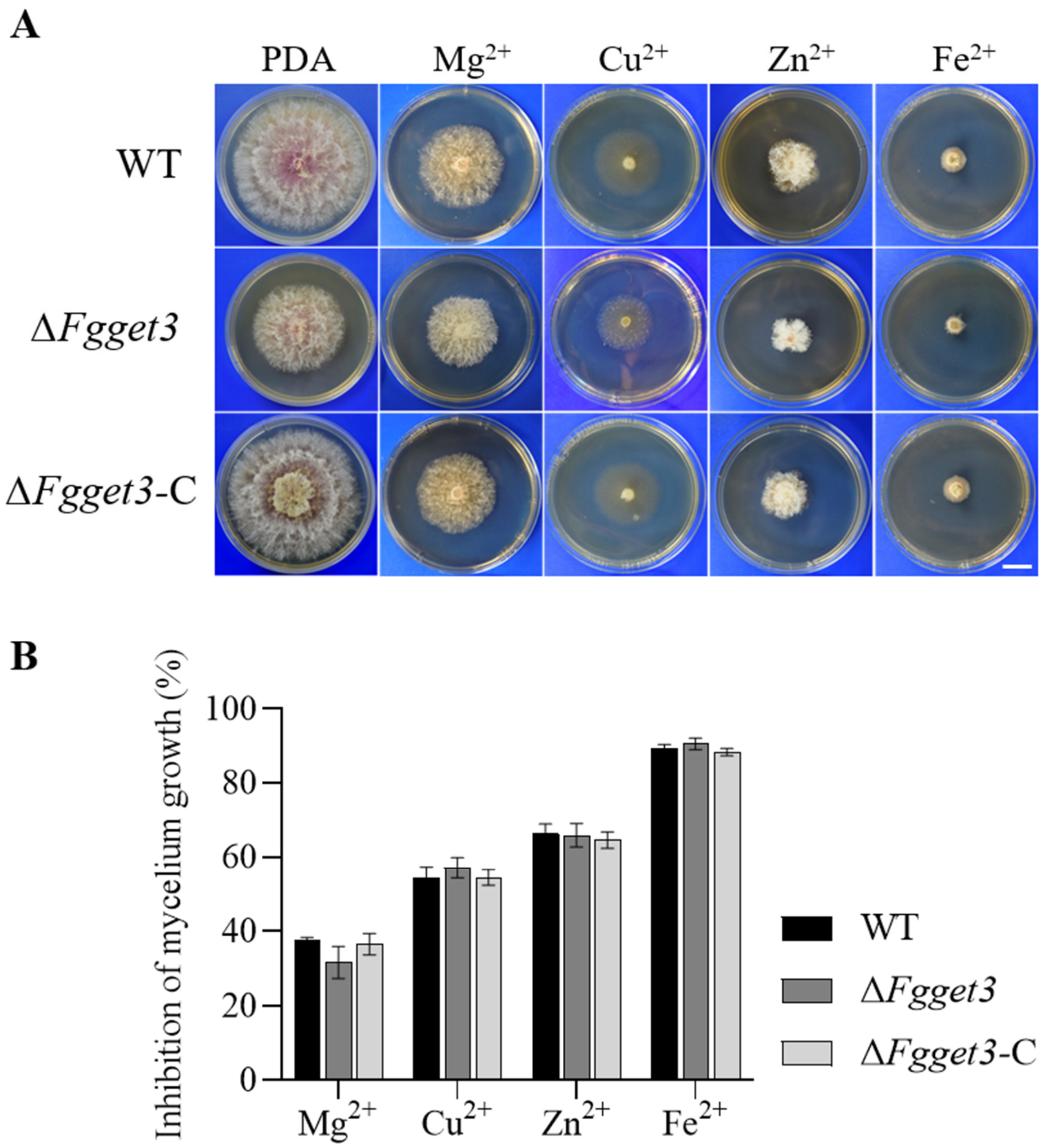

Figure S3 The responses of WT, Δ*Fgget3* and Δ*Fgget3*-C to metal stresses. (**A**) Colony morphology of tested strains in response to 0.4 M Mg^{2+}, 5 mM Cu^{2+}, 10 mM Zn^{2+} and 10 mM Fe^{2+}. Scale bar = 2 cm. (**B**) Percentage of mycelium growth inhibition of the tested strains. Means and standard deviations were calculated from three replicates (Student's t-test).

Table S1. Primers used in this study

Primer name	Oligonucleotide sequence (5'–3')	Remark
UP-F	ATTAAGGGAGTCACGAAGCTTGTG AGGTGAAGTGAGGCAGTG	To amplify the upstream sequence of *FgGET3* for the construction of the gene deletion
UP-R	ATCTAGAGGATCCCCCGACTAGTT TTGGCGGTGGAGGAGAAA	
D-F	AGGCCTGATCATCGATGGGCCCGC AAATGGGAGTATTATGTGG	To amplify the downstream sequence of *FgGET3* for the construction of the gene deletion
D-R	TAATTAAGTCCTCAGCGGCCCCT AGCGATTGTTTTGGTCTA	
M-UP-F	GTCTTACCCCTCCATCTTG	To identify *FgGET3* knock-out (KO) mutants
M-UP-R	TGCACTGTTTCTTCCTTGA	
M-D-F	TCTGAAATAAAGGGAGGAAG	To identify *FgGET3* KO mutants
M-D-R	AAACAAGCAATCTGGCAAC	
C-FgGET3-F	GAATGGCGAATGGAAATGCGGCC GAGTGTCAAGGACGTGACA	To amplify *FgGET3* with the native promoter used for the construction of the FgGET3-JM45 vector
C-FgGET3-R	TAGGGAAAGCTTGCATGCCTGCAG CTCGGGAGGAACATAAGGAG	
RT-FgGET3-F	TGGAAAGGGTGGTGTCGGA	To amplify *FgGET3* fragments used for the identification of the *FgGET3* KO mutants by RT-PCR
RT-FgGET3-R	ACGCAAACAAAGGTGGTGA	
HPH-F	TTCTGCGGGCGATTTGTGTA	To amplify the hygromycin resistance gene
HPH-R	TGACCTATTGCATCTCCCGC	
NEO-F	GCGGCGATACCGTAAAGCA	To amplify the neomycin resistance gene
NEO-R	ACTGAAGCGGGAAGGGACTG	
eGFP-FgGET3-F	GCTTGAGCAGACATCACCCGGGAT GTCCACCGCCCTCATCT	To amplify *FgGET3* fragments used for the construction of the pRFHUE-eGFP-FgGET3 vector
eGFP-FgGET3-R	CACCATTAAGTCCTCAGCCCCGGG CTCGGGAGGAACATAAGGAG	
pYES2-FgGET3-F	AACGGCCGCCAGTGTGCTGGAATT CATGTCCACCGCCCTCATCT	To amplify *FgGET3* fragments used for the construction of the pYES2-FgGET3 vector
pYES2-FgGET3-R	AATTACATGATGCGGCCCTCTAGA CTCGGGAGGAACATAAGGAG	

FgTUB2-F	GTTGATCTCCAAGATCCGTG	qPCR primers for analysis of *F. graminearum* DNA amount
FgTUB2-R	CATGCAAATGTCGTAGAGGG	
TaGAPDH-F	AACTGTTCATGCCATCACTGCCAC	qPCR primers for analysis of wheat DNA amount
TaGAPDH-R	AGGACATACCAGTGAGCTTGCCAT	
AD-FgGET3-F	GTACCAGATTACGCTCATATGATGTCCACCGCCCTCATCT	To amplify *FgGET3* fragments used for the construction of the AD-FgGET3 vector
AD-FgGET3-R	CAGCTCGAGCTCGATGGATCCCTCGGGAGGAACATAAGGAG	
BD-FgGET3-F	ATATGGCCATGGAGGCCGAATTCATGTCCACCGCCCTCATCT	To amplify *FgGET3* fragments used for the construction of the BD-FgGET3 vector
BD-FgGET3-R	TTATGCTAGTTATGCGGCCGCTGCAGCTCGGGAGGAACATAAGGAG	
